# Aerosolized Plus Intravenous Polymyxin B Versus Colistin in the Treatment of Pandrug-Resistant Klebsiella Pneumonia-mediated Ventilator-Associated Pneumonia: A Retrospective Cohort Study in Bangladesh

**DOI:** 10.2478/jccm-2023-0012

**Published:** 2023-05-08

**Authors:** Md Jahidul Hasan, Chandra Datta Sumi, Shihan Mahmud Redwanul Huq, Ahmad Mursel Anam, Raihan Rabbani

**Affiliations:** 1 University of New South Wales, Sydney, NSW, Australia; 2 The University of Queensland, Brisbane, Australia; 3 Internal Medicine and ICU, Square Hospitals Ltd., Dhaka, Bangladesh; 4 High Dependency Unit, Square Hospitals Ltd., Dhaka, Bangladesh

**Keywords:** polymyxin B, colistin, intravenous, aerosolization, Klebsiella pneumoniae, ventilator-associated pneumonia

## Abstract

**Background:**

Pandrug-resistant Klebsiella pneumoniae ventilator associated pneumonia (VAP) is associated with high rate of mortality in intensive care unit (ICU) and has been recognized as a difficult-to-treat infection worldwide. Polymyxin B or colistin-based combination therapies are frequently used worldwide though microbial eradication rate is not promising.

**Aim:**

The aim of this study is to compare the clinical outcome of intravenous with aerosolized polymyxin B versus colistin in the treatment of pandrug-resistant K. pneumoniae VAP.

**Methods:**

This retrospective cohort study was conducted on 222 mechanically ventilated patients admitted from May 11, 2019 to October 19, 2020. K. pneumoniae isolates were resistant to all available antibiotics, including polymyxins in culture sensitivity tests. As treatment, polymyxin B and colistin was administered in intravenous and aerosolized form concurrently twice daily in 106 patients and 116 patients in PMB and CLN group, respectively for 14 days. Survival rate, safety, and clinical outcomes were compared among the groups. The Cox proportional-hazard model was performed to calculate hazard ratio (HR) with 95% confidence intervals (CI).

**Results:**

Patients in PMB group showed more microbial eradication than the patients CLN group [68.1% (n=116)/83% (n=106), respectively; P <0.05). The median day of intubation and ICU stay in PMB group was shorter than that in CLN group [10 (IQR: 9-12.25) vs. 14 (IQR: 11-19), P <0.05; 12 (IQR: 10-14) vs. 15 (IQR: 9-18.5), P=0.072, respectively] with reduced 60-day all-cause mortality rate [15% (n=106) vs. 21.55% (n=116)]. Polymyxin B improved survival compared to colistin (multivariate HR: 0.662; 95% CI=0.359-1.222, P=0.195).

**Conclusions:**

Concurrent administration of intravenous and aerosolized polymyxin B in patients with pandrug-resistant K. pneumoniae-associated VAP revealed better microbial eradication, reduced the length of intubation and ICU stay, and improved survival rate compared to colistin.

## INTRODUCTION

An infection in the lung parenchyma preceded by invasive mechanical ventilation for at least a duration of 48 to 72 h at intensive care unit (ICU) setup is known as ventilator-associated pneumonia (VAP). VAP is one of the most common mechanical ventilator-associated infections in intubated critically ill patients and a difficult-to-treat infection among the nosocomial infections with high rate of mortality worldwide. VAP accounts for up to 29.1% of nosocomial pneumonia [1]. VAP causes hospitalization time longer, amplified treatment cost, and wastage of medical resources [2]. Despite advancements of microbiological techniques, standard diagnosis criteria and treatment of VAP is still a dueling issue which impedes the overall treatment process and abates clinical outcome in critically ill patients [1,2].

According to the clinical guideline of the Infectious Diseases Society of America (IDSA) and the American Thoracic Society (ATS) in 2016, up to 13% of patients die from VAP in each year [3], while another multi-center study showed that the 30-day mortality due to VAP, early VAP and late VAP was 29.9%, 19.2% and 31.4%, respectively in Europe [4]. A meta-analysis of 195 studies found that from 2006 to 2014 in China’s mainland, the cumulative VAP incidence rate was 23.8% with 22.83 patients per 1000 ventilator days [5]. A recent single-center study in Bangladesh found that 36.5% of mechanically ventilated patients in ICU developed VAP of which 78.9% and 21.2% was early and late VAP, respectively [6].

The gram-negative *Klebsiella pneumoniae*, a member of *Enterobacteriaceae* family, is one of the most common organisms for nosocomial pneumonia, including VAP around the world. The emergence of *K. pneumoniae* with pandrug (PDR)-resistant strains, resistant to all currently available antibiotics [7], is a big threat to global human health [8,9]. Polymyxin B and colistin (polymyxin E) are last-line polypeptide antibiotics intended to use for the treatment of multidrug-resistant *K. pneumoniae* infections, including VAP. Pandrug-resistant *K. pneumoniae* (PDRKP) strains frequently show resistance to polymyxins at usual doses and at that time, treatment of serious respiratory tract infections, including VAP becomes challenging [8–11]. To date, *in-vivo* data are limited regarding the pharmacokinetics of polymyxins in lung tissue after its intravenous or aerosolized administration and the susceptibility of PDR KP strains to polymyxins at differential drug-concentration in the lung tissue. Moreover, the paucity of data on polymyxins’ dose-mediated toxicity in patients with VAP develops conflicts regarding therapeutic modification [12,13]. The optimized therapeutic armamentarium with the last-hope polymyxins may be effective against the PDRKP strains causing VAP. A recent study showed that dual-routed (intravenous and inhalation) polymyxin B regimen exhibited better microbial eradication and increased survival rate compared to intravenous polymyxin B therapy in patients with VAP.

The objective of this study is to compare the ability of eradication of PDR *K. pneumoniae* and clinical outcome of polymyxin B with colistin while administering concomitantly in intravenous and aerosolized form in the treatment of pandrug-resistant *K. pneumoniae* VAP in critically ill patients in a tertiary care hospital in South Asia.

## MATERIALS AND METHODS

### Study design, participants and data collection

This was a single-centre retrospective cohort study conducted on 222 (N) mechanically ventilated adult patients (≥ 18 years) admitted from May 11, 2019 to October 19, 2020 in the ICU of a tertiary care hospital in Dhaka, Bangladesh. All the patients of the study with PDR *K. pneumoniae* VAP were taken into two different groups: (1) polymyxin B group (PMB group); and (2) colistin group (CLN group) where patients were treated with intravenous and aerosolized (concomitantly) polymyxin B (PMB) and colistin (CLN), respectively. Samples were distributed into PMB and CLN group following simple random sampling method. COVID-19 infection was assayed and confirmed in all patients at the time of hospital admission at the Emergency department by a positive reverse-transcriptase-polymerase-chain-reaction (RT-PCR) assay (instrument/device: Rotor Gene-Q/Cobas z480, and QIAGEN kits for real-time PCR, QIAGEN GmbH, Germany) of two specimens (nasal and oral swabs) collected by two trained phlebotomists following the hospital’s standard protocol of COVID-19 sample collection.

The PDR *K. pneumoniae* isolates was detected in tracheal aspirate or bronchoalveolar lavage in all patients of the study. The culture sensitivity test was performed by micro-broth dilution method using the instrument: BD Phoenix™ M50, BD Life Sciences: Diagnostics, USA). Identified *K. pneumoniae in study samples* were found confirmed resistant to all available antibiotics, including polymyxin B and colistin (Table 1), and the susceptibility parameters for colistin and polymyxin B were considered as sensitive: ≤2 mg/liter and resistant: >2 mg/liter according to the recommendations of Clinical and Laboratory Standards Institute (CLSI) and European Committee for Antimicrobial Susceptibility Testing (EUCAST), respectively [15, 16].

**Table 1.** Susceptibility of Klebsiella pneumoniae to all available antibiotics according to the CLSI^a^ and EUCAST^b^ guideline.

Antibiotics	Clinical breakpoints (μg/mL)	MIC (μg/mL)	Interpretation	Comment: Type of resistance
Amikacin	≤ 16, 32, ≥ 64	> 16	Resistant	Pandrug-resistant
Cefepime	≤ 2, 4-8, ≥ 16	> 16	Resistant	
Cefixime	≤ 1, 2, ≥ 4	> 2	Resistant	
Ceftazidime/Avibactam	≤ 8/4, ≥ 16/4	> 8/4	Resistant	
Ceftriaxone	≤ 1, 2, ≥ 4	> 4	Resistant	
Cefuroxime	≤ 4, 8-16, ≥ 32	> 8	Resistant	
Ciprofloxacin	≤ 1, 2, ≥ 4	> 1	Resistant	
Colistin	≤ 2, ≥ 4	> 2	Resistant	
Gentamicin	≤ 4, 8, ≥ 16	> 4	Resistant	
Imipenem	≤ 1, 2, ≥ 4	> 4	Resistant	
Levofloxacin	≤ 2, 4, ≥ 8	> 2	Resistant	
Meropenem	≤ 1, 2, ≥ 4	> 8	Resistant	
Piperacillin/Tazobactam	≤ 16/4, ≥ 28/4	> 64/4	Resistant	
Polymyxin B	≤ 2, ≥ 4	> 2	Resistant	
Tigecycline	≤ 1, 2, ≥ 2	> 2	Resistant	

a: Clinical and Laboratory Standards Institute;

b: European Committee for Antimicrobial Susceptibility Testing; μg: microgram; mL: milliliter; MIC: minimum inhibitory concentration

All the retrospective data of this study were collected from the electronic database and medical records of the hospital. The research related to human use has been complied with all the relevant national regulations, institutional policies, and under the tenets of the Declaration of Helsinki. This study was approved by the Research Ethics Committee, Square Hospitals Ltd, Dhaka, Bangladesh (no. 2101SH-OR02) on January 8, 2021. Written consent was taken from all participants in this study.

### Inclusion criteria

Sample inclusion criteria were as follows:

— patients with confirmed ventilator-associated pneumonia caused by pandrug-resistant *K. pneumoniae*
— no history of hospital admission, surgery, or development of pneumonia within the last four-weeks of hospital admission
— no history of sepsis or septic shock
— no other bacterial or fungal coinfection at the time of diagnosis of VAP

### Exclusion criteria

— history of COVID-19 infection within last 6 months or positive COVID-19 RT-PCR test during hospital admission
— patients shifted to the ICU from any other healthcare facility
— patients discharged from the ICU against medical advice or upon intubation
— evidence of concomitant bacterial or fungal respiratory tract infection during VAP treatment
— patients on hemodialysis or peritoneal dialysis, or therapeutic plasma exchange
— history of malignancy, obesity (pregnancy, and autoimmune diseases

### Diagnosis, definitions, and treatment

The pneumonia developed within forty-eight hours of endotracheal intubation was considered as ventilator-associated pneumonia. The International Classification of Diseases (ICD)-10 code of the diagnosed VAP infection was J95. 851.VAP was confirmed by chest computed tomography (CT) scan or X-ray having the presence of progressive pulmonary infiltrates with any two of the following symptoms: (1) persistent fever ≥100.4° F; (2) white blood cell count ≥11,000\mm3 of blood; (3) Purulent tracheobronchial secretions [17].

Pandrug-resistant isolate of bacteria is usually resistant to all commonly available antibiotics or even to reserve antibiotics, and identified through MIC test [7]. In this study, the PDR *K. pneumoniae* that caused VAP in intubated patients was fully resistant to polymyxins and not susceptible to any other antibiotic.

Patients in PMB group received polymyxin B (solution for injection) intravenously and in aerosolized form concomitantly. Intravenous dose and regimen: 20,000 units/Kg of body weight once at day one, and then 20,000 units/Kg of body weight/day divided into 2 equal doses (maximum 1.5 million units/day) from day two to fourteen.Aerosolized dose and regimen: 1 million unit/day divided into 2 equal doses.

Patients in CLN group received colistin (in the form of colistimethate Sodium solution for injection) intravenously and in aerosolized form concomitantly. Intravenous dose and regimen: 5 mg of colistin/Kg of body weight in a single dose at day one, and then 5 mg of colistin/Kg of body weight/day divided into 3 equal doses (maximum 300 mg colistin/day) from day 2 to 14. Each 33 mg of colistin (as base) is equivalent to 1 million units or 80 mg of colistimethate Sodium (CMS) [18]. Aerosolized dose and regimen: 99 mg of colistin/day divided into 3 equal doses. Along with polymyxin B or colistin therapy, usual dose of intravenous meropenem (adjusted in renal impairment) was given to all patients of both groups of the study.

Each intravenous and aerosolized dose of polymyxin B and colistin was administered in patients with 100 mL and 5 mL of 0.9% Sodium chloride solution over 1 h and 30 min, respectively. All the patients of both the groups (PMB/CLN) received aerosolized levosalbutamol over 30 min before receiving aerosolized dose of antibiotic (polymyxin B/colistin). The total duration of polymyxin B (PMB group) and colistin (CLN group) therapy was 14 days. All the patients in both groups received other medications according to the ICU’s treatment protocol. While creatinine clearance declined below 30 mL/min in patients of both groups, following intravenous doses of polymyxin B and colistin reduced to 50% of the total daily dose with same dosing frequency and aerosolized doses were remain unchanged. In case of bronchospasm, no antibiotic dose adjustment was done.

### Statistical analysis

The statistical analyses were performed by Statistical Product and Service Software (SPSS ver. 22.0, Chicago, IL, USA).All the tests were two-tailed. The categorical variables were analyzed between the treatment groups using Pearson’s chi-squared test. Continuous variables were compared Student’s t-test. Data were presented as numbers with percentage or medians with interquartile ranges (IQR), as appropriate. The hazard ratio (HR) with 95% confidence intervals (CI) was analyzed using the Cox proportional-hazard model. The multivariate model was adjusted for age, PaO_2_/FiO_2_ ratio, Acute Physiology and Chronic Health Evaluation (APACHE II), microbial eradication, intubation length, and length of ICU stay. The 60-day survival rate was assessed by Kaplan Meier (KM) plot using treatment groups (polymyxin B vs colistin) as factors; death as event and time to death as time variable. A *P* value less than 0.05 was considered statistically significant.

## RESULTS

Of 222 patients, 106 patients (N) and 116 patients (N) were included in PMB and CLN group, respectively. The Number of male patients was higher than that of female patients in both groups (74%/26%: PMB group and 53%/47%: CLN group), and the median age of patients in PMB and CLN group was 56 years (IQR: 44.5-68) and 55 years (IQR: 46-65), respectively. The body mass index value of all patients of the study was from 20 to 24. Comorbidities in all patients were recorded at the time of hospital admission and mentioned in Table 1. Before starting PMB and CLN therapy, the median peripheral capillary oxygen saturation in blood (SpO_2_), the median fraction of inspired oxygen (FiO_2_), and the median ratio of arterial oxygen partial pressure to fractional inspired oxygen (PaO_2_/FiO_2_) was 96% (IQR: 93-98)/96% (IQR: 94-98), 50 (IQR: 40-65)/45 (IQR: 40-65), and 240 mmHg (IQR: 193-255)/243 mmHg (IQR: 206.72-256) in patients of PMB and CLN group, respectively (*P*>0.05). Median values of blood components, infection markers, and liver enzymes in patients of both groups were mentioned and compared in Table 1. The median Modified Early Warning Score (MEWS) score and Acute Physiology and Chronic Health Evaluation (APACHE II) score was 4 (IQR: 3-4)/ 4 (IQR: 3-4) (*P*=0.032) and 20 (IQR: 20-24)/ 20 (IQR: 18-24) (*P*>0.05) in patients of PMB (N=106) and CLN group (N=116), respectively.

Patients treated with intravenous plus aerosolized polymyxin B in PMB group showed better microbial eradication (83%, N=106) than the patients treated with intravenous plus aerosolized colistin in CLN group (68.1%, N=116) (*P*<0.05). Secondary gram-negative bacterial infection within 21-day of detection of first PDR *K. pneumoniae* VAP infection was privileged more in CLN group than in PMB group, significantly (27.58% vs 22.64%, respectively). The median day of intubation and ICU stay was less in patients of PMB group than in CLN group’s patients [10 days (IQR: 9-12.25)/14 days (IQR: 11-19), respectively, *P*=0.001; 12 days (IQR: 10-14)/15 days (IQR: 9-18.5), respectively, *P*=0.072] (Table 2). Drug-induced adverse events were monitored continuously in every patient of both groups till discharged from the hospital. Patients who received aerosolized polymyxin B (PMB group) developed more bronchospasm (36.79%, N=106) compared to patients who received aerosolized colistin (CLN group) (6.89%, N=116) (*P*=0.001) but the incidence of nephrotoxicity was higher in CLN group compared to PMB group (28.44%/11.32%, respectively; *P* =0.002) (Table 3). Antibiotic therapy was continued after developing drug-induced bronchospasm in patients of the study and well managed with inhaled levosalbutamol as bronchodilator. Intravenous dose of polymyxin B and colistin was adjusted preceded by antibiotic-induced nephrotoxicity but therapy was not discontinued. Neurotoxicity associated with polymyxins was not found in any patient of the study.

**Table 2.** Baseline demographic information, comorbidity and laboratory findings in patients.

Variable	PMB group (N=106)	CLN group (N=116)	P value
Male/female, n (%)	78/28 (74/26)	62/54 (53/47)	
Age (year), median (IQR)	56 (44.5-68)	55 (46-65)	0.091
Fever (°F), median (IQR)	100.5 (100-101)	101 (100-101)	0.559
Diabetes, n (%)	89 (83.95)	97 (83.62)	0.022
Hypertension, n (%)	72 (67.93)	80 (68.96)	0.421
CVD, n (%)	61 (57.52)	53 (45.68)	0.542
Bronchial asthma, n (%)	27 (25.51)	30 (25.86)	0.031
CKD, n (%)	25 (23.63)	31 (26.72)	0.011
COPD, n (%)	25 (23.6)	27 (23.27)	0.058
Obesity, n (%)	22 (20.82)	18 (15.51)	0.316
PUD, n (%)	19 (17.94)	8 (6.89)	0.644
CLD, n (%)	9 (8.55)	12 (10.34)	0.021
PD, n (%)	4 (3.86)	9 (7.75)	0.465
SpO_2_ (%), median (IQR)	96 (93-98)	96 (94-98)	0.249
FiO_2_, median (IQR)	50 (40-65)	45 (40-65)	0.939
Respiratory rate, (breaths/min), median (IQR)	19 (16-24)	21 (18.29-24.7)	0.487
Heart rate (beat/min), median (IQR)	87 (83-97.37)	85 (78-90)	0.487
PaO_2_/FiO_2_ (mmHg) , median (IQR)	240 (193-255)	243 (206.72-256)	0.517
CRP (mg/L), median (IQR)	158 (136-268.3)	152.5 (125-243.81)	0.282
Procalcitonin (ng/mL), median (IQR)	5 (3.35-8)	6 (4.81-9)	0.114
WBC (K/μL), median (IQR)	18 (16.52-21)	16.46 (13.2-24)	0.001
Neutrophils (%), median (IQR)	65 (55.77-75)	75 (65-85)	0.454
Platelet (K/μL), median (IQR)	186 (151-263)	162 (125-242.5)	0.132
D-dimer (mg /L FEU), median (IQR)	0.96 (0.36-1.74)	0.57 (0.35-1.4)	0.005
LDH ((U/L), median (IQR)	419 (314-522)	425 (356.5-500.25)	0.117
Serum creatinine (mg/dL), median (IQR)	0.9 (0.7-1.2)	1 (0.8-1.6)	0.097
ALT (U/L), median (IQR)	56 (45-76)	57 (45-75)	0.597
AST (U/L), median (IQR)	48 (39-58)	46 (34-59)	0.813
Bilirubin (mg/dL), median (IQR)	0.8 (0.47-0.9)	0.8 (0.6-0.92)	0.737
MEWS, median (IQR)	4 (3-4)	4 (3-4)	0.032
APACHE II (score range: 0-71) , median (IQR)	20 (20-24)	20 (18-24)	0.171

IQR = interquartile range; n = number; % = percentage; °F = grade Fahrenheit; CVD = cardiovascular disease; CKD = chronic kidney disease; COPD = chronic obstructive pulmonary disease; PUD = peptic ulcer disease; CLD = chronic liver disease; PD = parkinson’s disease; SpO_2_ = oxygen saturation in blood; ; FiO_2_ = fraction of inspired oxygen; min = minute; PaO_2_/FiO_2_ = ratio of arterial oxygen partial pressure to fractional inspired oxygen; CRP = C-reactive protein; mg = milligram; L = liter; FEU = fibrinogen equivalent units; ng = nanogram; WBC = white blood cells; K/μL = thousand cells per micro liter; IL = interleukin; pg/mL = picograms per milliliter; LDH = lactate dehydrogenase; U/L = units per liter; dL = deciliter; ALT = alanine aminotransferase; AST = aspartate aminotransferase; MEWS = Modified Early Warning Score; APACHE II = Acute Physiology and Chronic Health Evaluation.

**Table 3.** Clinical outcomes in patients treated with intravenous and aerosolized polymyxin B or colistin

Parameters	PMB group (N=106)	CLN group (N=116)	P value
Microbial eradication, n (%)	88 (83)	79 (68.1)	0.001
21-day secondary bacterial infection, n (%)	24 (22.64)	28 (27.58)	0.013
Length-of-intubation (day), median (IQR)	10 (9-12.25)	14 (11-19)	0.001
Length-of-ICU stay (day), median (IQR)	12 (10-14)	15 (9-18.5)	0.072
Adverse drug event, n (%)			
Bronchospasm	39 (36.79)	8 (6.89)	0.001
Nephrotoxicity	12 (11.32)	33 (28.44)	0.002

PMB = polymyxin B; CLN = colistin; IQR = interquartile range; ICU = intensive care unit; n = number; % = percentage.

**Fig. 1.**
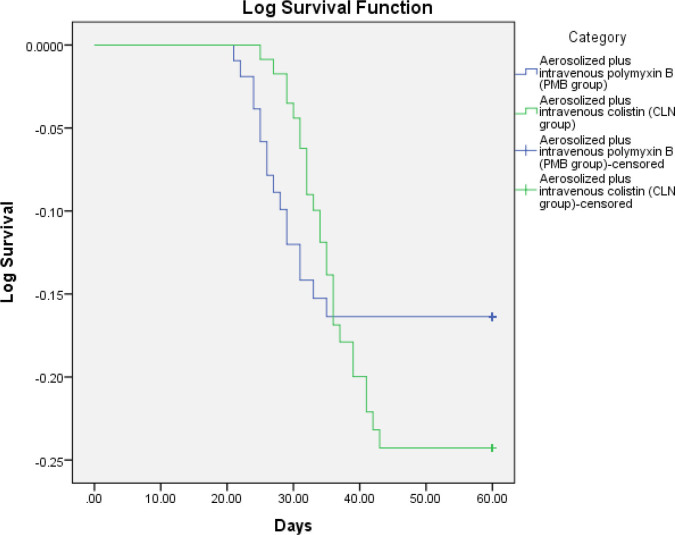
Kaplan-Meier survival curve for PMB group (aerosolized plus intravenous polymyxin B) (blue line) and CLN group (aerosolized plus intravenous colistin) (green line). Analysis run using Group (PMB vs CLN) as factor; death as event and time to death as time variable. Multivariate Hazard Ratio (H.R. for death: 0.035; 95% CI=0.007-0.168, *P*<0.001) is adjusted for baseline characteristics.

Sixteen deaths were observed in patients of PMB group (15%, N=106) while 25 deaths occurred in the CLN group (21.55%, N=116). The Cox proportional hazard model (adjusted for age, PaO_2_/FiO_2_ ratio, APACHE II, microbial non-eradication, length of intubation; and length of ICU stay) showed a significantly greater survival rate in PMB group as compared to CLN group (multivariate HR for death: 0.035; 95% CI=0.007-0.168, *P*<0.001) (Fig. 1). The survival rate did not differ with age, PaO2/FiO2 ratio, and length-of-ICU stay (multivariate HR: 1.028; 95% CI=1.001-1.055, *P* =0.045, 1.005; 95% CI=0.994-1.015, *P*>0.05, and 1.043; 95% CI=0.991-1.099, *P*>0.05, respectively). Non-eradication of PDR *K. pneumoniae* caused VAP significantly increased the risk of death (multivariate HR: 1.148; 95% CI: 0.484-2.721) in CLN group compared to PMB group. APACHE II and Length of intubation on mechanical ventilation was similar between the two groups, as shown in the Table 4.

**Table 4.** Results from the Cox Proportional Hazard model. GROUP=main variable (polymyxin B versus colistin).

Variable	HR	95% CI		P value
		Lower	Upper	
Group	0.035	0.007	0.168	<0.001
Age	1.028	1.001	1.055	0.045
PaO_2_/FiO_2_ ratio	1.005	0.994	1.015	0.379
APACHE II	0.958	0.862	1.063	0.415
Microbial non-eradication	1.148	0.484	2.721	0.038
Length of intubation	0.965	0.882	1.057	0.044
Length of ICU stay	1.043	0.99	1.099	0.117

HR=hazard ratio; CI =Confidence Intervals; CRP=C-reactive protein; PaO_2_/FiO_2_ = ratio of arterial oxygen partial pressure to fractional inspired oxygen; APACHE II = Acute Physiology and Chronic Health Evaluation; ICU = intensive care unit

## DISCUSSION

In this study, compared to colistin, concomitant intravenous and aerosolized polymyxin B exhibited better eradication of PDR *K. pneumoniae* with a significant reduction in intubation period and minimized the length-of-ICU stay in patients with PDR *K. pneumonia* VAP. A recent study on Bangladeshi population found that intravenous plus aerosolized polymyxin B eradicated 92.1% of multidrug-resistant (MDR) *K. pneumoniae* (only susceptible to polymyxin B) causing VAP [14] whereas in this study, the eradication rate of polymyxin B (intravenous plus aerosolization) was 83% while the *K. pneumoniae* strains were resistant to all available antibiotics including polymyxins.

The emergence of PDR gram-negative bacteria, such as *K. pneumoniae*, is currently a serious global public health concern [7–9]. Polymyxins are the reserve antibiotics for treating severe infections with MDR pathogens [8,9]. However, therapeutic outcome becomes very worse while *K. pneumoniae* isolates represent resistance to polymyxins [9–11]. VAP caused by PDR isolates of *K. pneumoniae* is most likely untreatable with the standard dose of polymyxin B or colistin given intravenously or aerosolized form [9,11]. Variable pharmacokinetic data of PMB and CLN have been demonstrated in different *in-vivo* studies to date [12,13,18,19]. The use of polymyxin B and colistin as last-line therapy in intravenous and aerosolized form in serious infections with MDR pathogens has been attributed in clinical practice over the last couple of decades [12]. In practice, in severe upper or lower respiratory tract infections, such as VAP, aerosolized polymyxins are currently more popular among the clinicians due to its outweighed therapeutic benefits over intravenous use [13,19,20]. However, clinical data related to the use of polymyxins in the treatment of PDR *K. pneumoniae* VAP mostly in South Asian population is limited [21].

Colistin is the bioactive form of CMS (administration form) and following an intravenous single dose of CMS, only 20% to 25% of the total CMS is rapidly converted to colistin-base activity (CBA) *in-vivo*, and the major portion of the drug is excreted through kidney as unchanged from of drug [20,22]. Following an intravenous dose of 150 mg CBA, the maximum plasma concentration (*C_max, Plasma_*) of colistin ranges from 0.4 to 0.77 mg/Liter of blood [22]. Imberti et al. [23] found that in critically ill patients, following intravenous 60 mg CBA every 8 h, at the steady-state phase, the formed colistin concentration was undetectable in bronchoalveolar lavage (BAL) fluid, and they also assumed that an increased intravenous dose may attribute a higher concentration of formed colistin in lung epithelial lining fluid (ELF) but nephrotoxicity would be the major concern. According to the findings of Athanassa et al. [24], an aerosolized 80 mg dose of CMS in mechanically ventilated patients resulted in high median CBA concentration (6.7 μg/mL) in ELF (locally converted) for up to 4 h after inhalation which met the MIC breakpoint for *K. pneumoniae* (<2 μg/mL). However, after 4 h, colistin concentration was declined below the MIC valueand a variety of sub-therapeutic colistin concentrations was found in BAL and ELFof the patients leading to therapeutic failure. A higher dose of inhaled colistin was suggested by the authors of the above study to achieve effective CLN concentration in the lung tissue for the eradication of MDR/PDR *K. pneumoniae* isolates causing serious RTIs, including VAP [24]. In this study, intravenous plus aerosolized dosage-regimen of colistin (total 400 mg CBA/day) successfully eradicated 68.1% of colistin resistant *K. pneumoniae* caused VAP and this may be due to high colistin concentration exposure to lung tissue and fluids.

In contrast, polymyxin B is administered as pharmacologically active sulfate salt form [19]. The *in-vivo* pharmacokinetic/pharmacodynamic data of intravenous or aerosolized polymyxin B is limited and the exact bioavailability of PMB in respiratory tract is difficult to predict. Yu-Wei et al. [25] in their Mouse Lung Infection Model found that after 5 min of an intravenous dose of 4.12 mg base (one milligram of pure polymyxin B base is equivalent to 10,000 units of polymyxin B sulfate)/kg of body weight of PMB, the average free plasma concentration of 1.07 ± 0.14 mg/liter was obtained and polymyxin B concentration in ELF following intravenous administration was below the ELF limit of quantification (LOQ) (~2.00 mg/liter) due to low rate of free-drug transfer from plasma to ELF (CL_Plasma, ELF_ of 1.14 × 10^−4^ liters/h/kg) which does not meet the required MIC level (2 μg/mL as per CLSI) in ELF essential for killing PDR *K. pneumoniae* in lung. On the other hand, following pulmonary administration of 4.12 mg base/kg of body weight of PMB, the maximum achieved ELF concentration (*C_max, ELF_*) was 107.0 ± 24.6 mg/liter and the concentration was significant over 12 h meeting the MIC breakpoint of 2.00 mg/liter. Thus, 12-hourly aerosolized plus intravenous dosages of PMB may ensure a significant PMB level in lung tissue and fluids over 24 h above the MIC level, and with this dual dosage-regimen in the study, we found significant eradication of PDR *K. pneumoniae* with polymyxin B (83%, N=106). In a recent study on South Asian population, intravenous plus aerosolized PMB resulted in higher eradication of MDR *K. pneumoniae* compared to intravenous PMB (92.1% vs 70.1%; *P*<0.05) [14]. Likewise, in our study, PMB exhibited superior microbial eradication rather than colistin in similar dual-route administration model (83% vs 68.1%, P<0.05).

*K. pneumoniae* exhibits resistance to polymyxins through developing chromosomal mutation or horizontal gene-transfer of plasmid which reduce the binding affinity of polymyxins to bacterial outer cell membrane required for increased membrane permeability leading to bacterial death [26]. The exact mechanism behind the ability of polymyxin B and colistin at high exposed concentration to kill polymyxins resistant *K. pneumoniae* is still not known. However, in our study, through concomitant intravenous plus aerosolized administration, a relatively higher concentration of polymyxin B and colistin might be attained in the lung but, compared to colistin, PMB showed better microbial eradication, decreased length-of-intubation and ICU stay, and increased survival rate. Following intravenous or inhaled dose, colistin concentration in lung tissue and ELF was found insignificant (below MIC breakpoint) in time-response while polymyxin B showed sustainable drug level and maintained MIC level in the infected lung [23,24]. Considering these findings in previous studies, our study found better recovery and survival with concomitant intravenous and aerosolized polymyxin B compared to colistin in patients with PDR *K. pneumoniae* VAP. In addition, the multivariate HR for death was significantly more in CLN group compared to PMB group (0.035; 95% CI=0.007-0.168). May be due to high PMB concentration in lung in PMB group’s patients, incidence of bronchospasm was higher in PMB group than the CLN group but, high exposure of colistin in kidney in patients of CLN group developed more nephrotoxicity.

To determine the actual pharmacokinetics and pharmacodynamics of polymyxin B and colistin in post intravenous plus aerosolized administration and the definite mechanism of high polymyxins concentration-mediated killing of pandrug-resistant *K. pneumoniae* in patients with VAP, a large randomized control trial is highly required during the emergence of highly resistant *K. pneumoniae* infections worldwide. No genetic identification of PDR *K. pneumoniae* strains, no pharmacokinetic assessment of intravenous or aerosolized polymyxins in plasma or lung, and lack of pharmacokinetic evidences of inhaled dosages of polymyxins were the major limitations of this study.

## CONCLUSION

Pandrug-resistant *K. pneumoniae* VAP is a serious life-threatening infection with limited effective antibiotic option. This study found better microbial eradication, reduced length-of-intubation and ICU stay, and improved 60-day survival rate in patients with pandrug-resistant *K. pneumoniae* VAP treated with intravenous and aerosolized polymyxin B concomitantly compared to colistin.
